# Trauma, Mental Health Distress, and Infectious Disease Prevention Among Women Recently Released From Incarceration

**DOI:** 10.3389/fpsyt.2022.867445

**Published:** 2022-05-20

**Authors:** Karen A. Johnson, Timothy Hunt, Lisa B. Puglisi, Daniel Maeng, Amali Epa-Llop, Johanna E. Elumn, Antoinette Nguyen, Ashley Leung, Rachel Chen, Zainab Shah, Jiayi Wang, Rachel Johnson, Benjamin P. Chapman, Louisa Gilbert, Nabila El-Bassel, Diane S. Morse

**Affiliations:** ^1^University of Alabama School of Social Work, Tuscaloosa, AL, United States; ^2^Social Intervention Group, Columbia University School of Social Work, New York, NY, United States; ^3^SEICHE Center for Health and Justice, Yale School of Medicine, New Haven, CT, United States; ^4^Section of General Internal Medicine, Yale School of Medicine, New Haven, CT, United States; ^5^Department of Psychiatry, University of Rochester School of Medicine, Rochester, NY, United States; ^6^Department of Medicine, University of Rochester School of Medicine, Rochester, NY, United States

**Keywords:** psychopathology, trauma, PTSD, HIV, hepatitis C, sexually transmitted infections, recently released women

## Abstract

**Background:**

U.S. women recently released from incarceration experience significantly higher rates of trauma and exacerbation of mental health conditions, and the period following release has been identified as a window of heightened risk for mental health distress and human immunodeficiency virus (HIV), sexually transmitted infections (STI) and hepatitis C (HCV) transmissions. Despite these vulnerabilities, and an urgent need for supports, optimal engagement strategies remain unclear. WORTH Transitions is a program made up of two evidence-based interventions focused on improving the health of women returning to the community from incarceration with substance use disorders. Combining the two was designed to reduce HIV/STIs/HCV risks and increase overall health treatment engagement using a community health worker led intervention.

**Methods:**

We examined associations between trauma, mental health symptomology, and HIV/STI/HCV outcomes among women who engaged in the WORTH Transitions intervention (*N* = 206) Specifically, bivariate and longitudinal multivariate models were created to examine associations between trauma and mental health distress (defined as depressive and PTSD symptoms), on (1) types of engagement in HIV/STIs/HCV prevention and behavioral health services; and (2) HIV/STIs/HCV risk outcomes. The women who engaged in the intervention were 18 years and older and some were White, Black and other racial or ethnic minority.

**Results:**

PTSD symptomology and being a Black or indigenous woman of color was significantly (*p* = 0.014) associated with individual or group session engagement. Neither trauma nor PTSD symptoms were associated with higher HIV/STIs/HCV risks. Instead, relative to those who did not engage in HIV/STI/HCV risky behaviors, PTSD symptomology (*p* = 0.040) was associated with more than 3-fold increase in the probability of being lost to follow up (relative risk ratio = 3.722).

**Conclusion:**

Given the impact of PTSD-related symptoms on driving both engagement in HIV/STIs/HCV prevention services and intervention attrition among women leaving incarceration, physical and behavioral health interventions must be both overtly trauma- and mental health-informed. As was the case with WORTH Transitions, physical and behavioral health services for this population must include intentional and active support of the forms of treatment participants endorse to ensure maximal engagement.

## Introduction

U.S. women recently released from jails and prisons are subject to significantly higher levels of trauma and mental health distress such as post-traumatic stress disorder (PTSD) and depressive symptomology (1). Personal and interpersonal forms of trauma experienced post-incarceration include ones related to adult victimization (2), family (3), alcohol and substance misuse (4), violence from intimate, casual, and paying sexual partners (5), and risky sexual encounters (e.g., transactional sex, condomless sex, sex while high, and/or sex with partners that are known HIV positive) (6, 7). Rates of intimate partner violence as a whole have been found to be ten times levels found in the general population (8), with physical violence as high as 25 percent within the first 6 months after release. This is roughly five to six times higher than among the general population (9). Depressive symptomology and lifetime PTSD prevalence are also high at 44.6 (10) and 53 percent (11), respectively, compared to 12 (12) and 6 (13) percent in the general population. Pervasive levels of trauma are also associated with criminal legal system involvement itself (14) and social and structural determinants of health such as poverty, un- and under-employment, housing insecurity and homelessness, and healthcare stigma (15).

Alongside these significant associations, accumulated trauma and mental health conditions among recently released women are associated with disproportionately high rates of HIV, other sexually transmitted infections (STIs) and Hepatitis C (HCV) (11, 16–19). HIV transmission rates alone are three times levels found among US adults (1.3 vs. 0.5%) (20). The existing literature collectively show high prevalence of exposure to traumatic events and/or recent PTSD across a variety of categories (including childhood and lifetime physical and sexual abuse) among HIV-positive women (21). Severe trauma exposure among women has been associated with HIV risk behaviors such as greater numbers of sexual partners and engagement in transactional sex/sex-trading (22). In addition to domestic violence and sexual victimization, experience of community violence (measured as exposure to violent crime) has also been found to predict sexual risk behaviors among a sample of HIV-positive women (23). Importantly, greater trauma exposure is linked to poor treatment adherence and poor HIV-related health outcomes (24).

Given these intersecting risks, the period following release from incarceration has been identified as a particularly vulnerable window of re-traumatization (25) and HIV/STI/HCV risk behavior re-engagement (26–28). It has also been identified as an opportune time for targeted health and behavioral health treatment interventions (29). And yet, some of these very risk factors (high rates of trauma, mental health symptomology and other comorbid risks such as substance misuse, etc.) have been identified as significant barriers to care (30). Recently released women can be among the hardest to engage in HIV/STIs/HCV prevention services, particularly during the year following release (31). The need for targeted intervention is made especially urgent by steep inclines in the incarceration rates for women in the United States (32, 33). Almost 1.8 million women are released from jails each year and an additional 81,000 transition from state prisons (34). Paradoxically, although ~80% of women transitioning from jails and prisons report chronic medical, psychiatric, or substance abuse problems, only 15% reported visiting a physician outside of the emergency department in the first-year post release for physical and/or behavioral health care (35). Perceived lack of engagement with physical and behavioral health treatment, such as chronically missed appointments and low adherence to medication regimens, has been linked to having underlying mental health conditions, such as depression and PTSD, and to exposure to traumatic life events (36, 37).

Despite these intersectional risks, and the well-established efficacy of trauma-specific care (38), a significant gap remains regarding optimal trauma-informed physical and behavioral health engagement strategies needed to promote participation in HIV/STIs/HCV prevention services among women recently released from incarceration (32, 39). Similarly, little research has examined accumulated trauma and mental health distress (defined as PTSD and depressive symptoms) on HIV/STI/HCV prevention and treatment engagement, and HIV/STIs/HCV risk outcomes among women released from incarceration. Further complicating this challenge, findings (where available) are somewhat contradictory. Results to date suggest that highly traumatized women transitioning from incarceration possess both higher levels of treatment hesitancy and health- and treatment-seeking behaviors (40). PTSD is associated with low engagement in care among male and female populations transitioning from jail (41). Conversely, depressive symptomology is significantly associated with greater (not less) interest in a pre-exposure prophylaxis (PrEP) intervention (42). Given these associations between incarceration, accumulated trauma, and both depressive and post-traumatic stress symptomology, these latter findings suggest that women transitioning from jails and prisons may encounter even greater difficulty when attempting to adhere to HIV treatment and engage in care.

In view of these significant gaps, and contradictory findings where available, additional research is needed to extend understanding of associations between trauma, mental health, treatment engagement, and HIV/STIs/HCV outcomes among recently released women to guide efforts to prevent infectious diseases in this especially vulnerable population. To test the hypothesis, we looked at whether trauma has any independent impact on engagement type and/or the likelihood of engaging in risky sexual behaviors. For those who did not complete the study, we examined the impact of trauma on the likelihood of responding to the 6-month survey. Leveraging 6-month longitudinal data collected from cis-gender women (*n* = 206) participating in the WORTH Transitions program, all of whom transitioned from incarceration within 12 months of enrollment in the study, associations were examined between forms and amount of trauma experienced, depressive and PTSD symptomology, and study engagement and HIV/STIs/HCV risk behaviors. Given U.S.' commitment to end the HIV epidemic among marginalized populations and increased reliance on probation, parole, and other forms of community corrections, this study fills a significant research and public health gap. Deepening understanding of these heretofore understudied associations is critical in guiding efforts to sharpen current efforts to address this ongoing public health risk in this highly traumatized population during the especially vulnerable period post-release.

## Methods

### Intervention

WORTH Transitions is a multi-site, HIV/STI/HCV prevention and intervention study for high-risk women recently released from incarceration that integrates two evidence-based programs: Women on the Road to Health (WORTH) as well as the version adapted for Black women specifically (E-WORTH: Empowering African-American Women on the Road to Health) and Transitions Clinic. WORTH (43) and E-WORTH (44) is a structured, five-session, multimedia intervention, efficacious in decreasing HIV/STI risks, intimate partner violence, and substance use among women involved in the criminal legal system. WORTH Transitions adaptation was tailored for recently released women, which included specifically addressing trauma experiences. Transitions Clinic is part of a national network of primary care-based programs that focus on the health needs of people returning to the community from incarceration by centering peer community health workers (CHWs) with their own history of incarceration to help patients engage with the health system and address social determinants of health (45). Engagement with Transitions Clinic has been found to reduce acute care utilization, hospitalization for illnesses preventable with access to primary care, and criminal legal system contact (35, 46). A growing body of literature has also demonstrated the effectiveness of peer CHWs in helping to increase access to needed HIV and other prevention and intervention services, while minimizing stigma (47, 48). The use of peer community health agents has also been linked to a decrease in the level of stigma in typical healthcare-related interactions that can interfere with addressing HIV and other STIs, HCV and concomitant risks of substance use disorder and intimate partner violence (49).

WORTH Transitions was adapted to be more trauma-informed, therefore we expect both the transitions clinic and the WORTH sessions to alleviate PTSD symptoms. However, the literature on this population is scarce. Of the available research that exists, it is possible that competing needs may influence their choices of engagement (50). For example, if participants have urgent health care needs they may be more likely to engage in healthcare only. Additionally, some literature does suggest that group sessions, especially with peers, are helpful in working through traumatic experiences and may yield more positive results in those with histories of trauma in need of socialization and support (30). More research is needed to understand these differences.

### Study Team

WORTH Transitions was made possible from a collaboration of University of Rochester School of Medicine, Yale University School of Medicine, and Columbia University School of Social Work Social Intervention Group. The University of Rochester Institutional Review Board and then those at the other two institutions approved this study (#00001140).

### Recruitment and Enrollment

Participants were recruited, consented, and screened by research assistants or formerly incarcerated community health workers from jails and prisons and multiple other community locations at which recently released women frequent and receive services (e.g., women's transitional housing, homeless shelters, food pantries, public defender offices, courthouses, probation, and substance use disorder treatment programs) in Rochester, New York and New Haven, Connecticut. Indirect recruitment methods were also implemented using social media ads (e.g., Facebook) and flyers posted in frequented locations. Participants could receive a total of $250 in gift card incentives to participate in the study in the following intervals: $10 for screening; $25 for baseline interview; $20 for attending individual and each of the four group WORTH sessions which included HIV/HCV testing in the individual session; $10 for each monthly check-in; $20 for giving a written or video testimonial for our Facebook page; and $30 for 6-month interview.

In addition to being female (cis- or transgender) and recently released from incarceration within 12 months prior to recruitment, inclusion criteria included: English-speaking, being 18 years and older, self-reported recent alcohol and/or substance use disorder histories, agreement to research requirement related to (a) allowing access to medical/mental health/SUD (Substance Use Disorder) treatment records during their participation in the study; and (b) accessing physical and behavioral health treatment. Being able to read was not required. Exclusion criteria included cognitive vulnerability (e.g., scoring <2 on the Six-Item Screener derived from the Mini-Mental Status Examination) (51), and/or declining HIV/STI/HCV assessment. There were no selection criteria related to race and ethnicity.

A total of 259 women were screened, 208 enrolled (110 from Rochester; 98 from New Haven), and 206 completed baseline measures. Full participation in the study was defined as completion of the study's initial screening, an individual (1:1) WORTH HIV/STI/HCV prevention session including HIV/HCV rapid test and pre/post-test counseling, 4 group sessions, and at least 1 primary care provider (PCP) appointment attended at either the WORTH Transition clinics or their own PCP in Rochester or New Haven. Further HIV/STI/HCV testing and treatment were administered during PCP appointments as required. WORTH Transition personnel and clinic staff received training in trauma-informed care prior to the launch of the study. Participants were referred to substance use and mental health counseling services when such services were deemed clinically necessary or upon participant request.

While participants were encouraged to complete all study activities, as much engagement as participants found comfortable was allowed in view of the high amount of trauma anticipated in the population. In addition, participants who elected to receive physical and behavioral health services from a PCP outside of Transitions Clinic were supported in their decision to do so to minimize trauma associated with a disruption in care. To promote maximum recovery, alternative forms of support were also encouraged such as Twelve Step and other forms of self-help, support groups, or spiritual counseling. WORTH individual and group sessions were delivered by trained, formerly incarcerated CHWs. Outreach activities were also primarily conducted by peers. Transition Clinic appointments were conducted by a lone study PI/co-I at each intervention site trained in trauma-informed, trauma-specific, and culturally-informed care as part of routine care.

## Measures

All explanatory variables (Trauma, Intimate Partner Violence, and Depression symptomology) were collected at baseline. Outcome variables (Engagement, HIV/STI/HCV risks) were collected during subsequent time points. Engagement was defined as participating in individual and/or group WORTH sessions and/or receiving clinical services from the Transition Clinic post baseline. HIV/STI/HCV risk variables were collected at the 6-month survey timepoint.

### Explanatory Variables

#### Clinical Assessments

##### Trauma

###### Traumatic Events.

Trauma was modeled in two ways using the Lifetime Stressors Checklist-R (LSC-R) (52). The LSC-R is a 30 item self-report measure that assesses traumatic events over the course of the lifecycle. Items include traumatic experiences with physical abuse/assault, sexual abuse/assault, and others related to medical-, financial-, relationship-, and family-trauma. Questions follow a yes/no response format. In line with previous research (53), a summary variable was computed based on the sum of positive answers in the LSC-R. This computed score most directly reflects breadth of trauma exposure (i.e., higher scores indicate that a participant has experienced many types of traumas); the variable purposefully does not attempt to assign trauma exposures different weights, and thus may not reflect trauma accumulation. As there is no agreed-upon index of trauma severity based on the LSC-R, measuring breadth of trauma exposure seemed to be a reasonable quantification of LSC-R responses.

For the purposes of this study, the item pertaining to experiences with natural disasters was eliminated. In this study, the LSC-R was examined first as a composite variable with values ranging from 0 to 29. A score of 29 indicates that the participant experienced all of the events. Second, we recoded some items of the LSC-R into clusters of trauma types, the first five of which were coded as continuous variables: physical abuse/assault, sexual abuse/assault, family, medical, and system. The remaining four clusters were modeled as binary, single item variables: emotional abuse, financial, relationship, and other events: (e.g., “Are there any events we did not include that you would like to mention?”).The LSC-R has been used with criminal legal system populations and has moderate to good test-retest reliability, and good convergent and criterion validity with PTSD symptoms in HIV infected women (54).

###### Intimate Partner Violence.

Intimate partner violence was modeled using the 5-item, yes/no, self-report Ongoing Abuse Scale (OAS) (55) measuring physical (e.g., “Are you presently emotionally or physically abused by your partner or someone important to you?”), sexual (e.g., “Are you presently forced to have sexual activities?”), and emotional intimate partner violence at the present time or presently. The OAS has been tested with women, African Americans, Latino/as and Whites in various settings (Chronbach alpha of 0.59) (55).

##### Psychopathology

###### Depressive Symptomology.

Depressive symptomology, measured at each time point, was modeled as an ordinal/categorical variable using the *Global Assessment of Individual Needs* (*GAIN)* scale, which is a 113-page validated assessment, usable in shorter subsections, for detailed treatment planning and monitoring of individuals with SUD, yielding a composite score of need, access, and utilization. The reliable and well-validated GAIN Internal Mental Distress Scale (IMDS) component assesses depression and anxiety symptoms over the preceding 90 days and year (severe, none or moderate) (56).

###### Post-traumatic Stress Disorder Symptoms.

PTSD was modeled as a dichotomous variable (negative, positive) using the Trauma Symptom Scale component of the IMDS which reliably measures PTSD (α = 0.96).

### Covariates

#### Sociodemographics

The following sociodemographic variables were included in the model: (a) relationship status (single, in relationship, separated/divorced/widowed); (b) age (Under 30, 30–40, over 40), which was chosen based on the distribution of the data (c) educational level [less than High School (HS), HS/General Educational Development (GED), some college, college+]; (d) race/ethnicity (white, non-white); (e) homelessness status (homeless, not homeless); (f) employment status (unemployed or under, employed full- or part-time, unknown/not reported); and (g) ever in foster care or adopted (no, yes).

##### External Support Accessed

Modeled as a dichotomous variable, external support accessed during their time in WORTH Transitions was included in the model using a single variable [attended self-help group, attended religious program(s), attended recovery program(s), attend any of the above].

#### Clinical Measures

##### Alcohol Use

Alcohol use was modeled as the number of days out of past 90 days during which the patient used alcohol, coded as the following categories: 0 day, 1–30 days, and >30 days.

##### Substance Use

Substance abuse was modeled as a count of substance used in the past 90 days out of a total of 13 substances (e.g., marijuana, crack, cocaine, heroin, methadone, PCP, hallucinogen, crystal meth, speed, anti-anxiety, downers, painkillers) (none, 1–3, 4 or more -max 8).

#### Non-clinical Measures

##### Time Since Released

Using a single item question (“How long has it been since you were released from incarceration?”), time since released from jail or prison was modeled as a categorical variable with four discrete categories: <1 month, 1–3 months, 3–6 months, and >6 months.

### Outcome Variables

#### Engagement

“Engagement” was defined as a categorical variable with four discrete types of possible engagement in WORTH Transitions programming: (a) choosing not to participate in individual or group WORTH sessions or attend Transitions Clinic (“no engagement”); (b) choosing only to participate in WORTH individual or group sessions (“session only”); (c) choosing only to participate in Transitions Clinic services and no WORTH sessions (“healthcare only”) (d) choosing to access both WORTH sessions and Transitions Clinic/primary care physician services (“full engagement”).

#### HIV/STI/HCV Risks

Measured at the 6-month mark, HIV/STI/HCV risk outcomes were modeled as a composite variable using self-reported risk behaviors (unprotected sex, risky needle use, sex with someone who injects drugs, sex while high, sex with a male sexual partner who was HIV+) and/or as self-reported HIV/STI/HCV. Any risk behavior endorsed and/or self-reported HIV/STI/HCV was assigned a maximum of one point such that participants who endorsed all risk behaviors and indicated that they received positive diagnosis received a total of one point. Similarly, those reporting only one risk behavior or self-reported HIV/STI/HCV, received a total of one point.

Risk behaviors were measured using the GAIN HIV risk scale (α = 0.96).

Self-reported HIV/STI/HCV was measured using yes/no responses to the question: “In the past 6 months, have you been diagnosed with a sexually transmitted disease/HIV/Hep C?” (56).

### Statistical Analyses

All statistical analyses were performed using STATA version 15.1 statistics software package.

#### Bivariate Analyses

##### Engagement Type

Types of engagement (“engagement none”, “engagement: session only”, “engagement: healthcare only”, and “engagement: full engagement”) was similarly analyzed in bivariate analyses on sociodemographic variables (relationship status, age, educational level, race/ethnicity, homelessness status, employment status, ever in foster care or adopted), key variables of interest (trauma, mental health: depressive symptoms, PTSD symptoms) and all other variables in the study (time since release, external support, number of days alcohol used during the past 90 days, count of substance used). Bivariate analyses were carried out via chi-square test of independence for discrete variables and ANOVA for continuous variables.

##### HIV/STI/HCV Risks

Stratified HIV/STI/HCV risk behavior categories: “no risky behavior”, “any risky behavior”, and “no 6-month data”, were examined in bivariate analyses with engagement type, key variables of interest, controlling for sociodemographic variables and all other variables in the study.

#### Longitudinal Multivariate Analyses

##### Engagement Type

Adjusted (model 1a) and unadjusted (model 1a-U) longitudinal multivariate models were built to examine associations between baseline trauma (forms and accumulated), baseline mental health conditions on types of engagement endorsed (no engagement vs. session only; no engagement vs. healthcare only; no engagement vs. full engagement), controlling for all other variables in the study. The accumulated “trauma score” is a continuous variable that can range from 0 to 29. X_i_ represents the set of control variables, which include the following: whether the patient had any external support (=1 if she attended one or more of self-help group, religious group, or recovery org and 0 otherwise), minority status, screened for severe depression symptoms, screened for PTSD symptoms, relationship status, ever in foster care or adopted, education level, age, time since prison release, count of substances used during the past 90 days. For details on how each of these variables were operationalized in the analysis, refer to the attached XL file. For model 1a [engagement type_i_ = a_1_ (Trauma Score_i_) + a_2_ (X_i_) + e_i_], “engagement type” can take 4 discrete outcomes (no engagement vs. session only vs. healthcare only vs. full engagement). Since there are 4 discrete outcomes, Model 1a is estimated via a multinomial logistic regression model, resulting in 3 separate sets of results, using “no engagement” as the reference category: (1) no engagement vs. session only, (2) no engagement vs. healthcare only, and (3) no engagement vs. full engagement. Unadjusted model 1a (Model 1a-U): Engagement_i_ = α_1_(Trauma Score_i_) + e_i_.

##### HIV/STI/HCV Risks

Finally, adjusted [Model 2a: RiskyBehaviors_i_ = c_1_ (Trauma Score_i_) + c_2_ (Engagement type_i_) + c_3_ (X_i_) + e_i_] and unadjusted models [(Model 2a-U) RiskyBehaviors_i_ = γ_1_ (Trauma Score_i_) + δ_2_ (Engagement type_i_) + e_i_] were built to examine longitudinal associations between trauma, depressive and PTSD symptomology, engagement type, and HIV/STI/HCV outcomes. Where risky behaviors_i_ (RiskyBehaviors) can take 3 discrete outcomes as the following: None, Any, or No 6-month data, model 2a is estimated using a multinomial logistic regression model to test the hypothesis that either trauma or engagement type has any independent impact on the likelihood of engaging in risky sexual behaviors (and also on the likelihood of responding to the 6-month survey).

## Results

### Sample Characteristics

Study participants were disproportionately Black and indigenous women of color (BIWOC) (45.6%), single (55.3%), over 30 years (78.1%), 32.5% of which were 40 and older. Sample characteristics also included high rates of recent homelessness (38.8%), current under- and unemployment (86.4%), recent substance use (59.8%) and non-study help seeking behavior (e.g., self-help groups) (66.5%). Participants were also on the whole evenly distributed across the four time periods since release. Eighty two percent of participants reported lifetime intimate partner violence.

The mean trauma score was 13.9 (out of a maximum of 29). Financial abuse (83%) and emotional abuse (74%) were the most heavily endorsed sub-trauma domains. Women also reported experiencing a high degree of system-trauma (1.18 out of a total possible score of 2), physical abuse/assault trauma across the lifecycle (2.42 out of 5) and medical trauma (1.95 out 2).

Close to three-quarters of women in the sample were identified as having depression symptomology (72.8%) and an even higher amount endorsed post-traumatic stress disorder symptomology (82.0%).

### Bivariate Analyses

#### Engagement Type

Table 1 presents findings of descriptive statistics and bivariate comparisons examining forms of engagement (“engagement none”, “engagement: session only” “engagement: healthcare only”, and “engagement: full engagement”) on sociodemographic variables and study controls. Being BIWOC was significantly associated with visits to the Transition Clinic/receiving health care services. Being un- or underemployed and using alcohol in excess of 30 days was significantly associated with participating in sessions only. Experiencing intimate partner violence was significantly associated with engagement in all study activities (Transition Clinic or PCP and Sessions) whereas experiencing lifetime physical abuse/assault and the overall trauma score was associated with engaging in sessions only.

**Table 1:** Descriptive statistics by engagement type.

**Patient characteristics**	**All sample**	**No engagement**	**Session only**	**Healthcare only**	**Full engagement**	**chi-square test of independence (*p*-value)**	
N	206	39	85	20	62		
**Race/ethnicity**							
White	53.9%	69.2%	51.8%	35.0%	53.2%	0.081	
Minority	45.6%	28.2%	48.2%	65.0%	46.8%	0.045*	
Missing	0.5%	2.6%	0.0%	0.0%	0.0%	0.231	
**Homelessness**							
Not homeless	61.2%	51.3%	67.1%	60.0%	59.7%	0.405	
Homeless	38.8%	48.7%	32.9%	40.0%	40.3%	0.405	
**Depression**							
Severe	72.8%	66.7%	75.3%	70.0%	74.2%	0.765	
None or moderate	24.3%	28.2%	22.4%	25.0%	24.2%	0.918	
Missing	2.9%	5.1%	2.4%	5.0%	1.6%	0.694	
**PTSD**							
Negative	17.5%	30.8%	12.9%	25.0%	12.9%	0.053	
Positive	82.0%	69.2%	87.1%	75.0%	85.5%	0.073	
Missing	0.5%	0.0%	0.0%	0.0%	1.6%	0.506	
**Time since release**							
<1 month	23.3%	23.1%	30.6%	10.0%	17.7%	0.134	
1–3 months	29.1%	41.0%	17.6%	20.0%	40.3%	0.005*	
3–6 months	19.4%	20.5%	14.1%	25.0%	24.2%	0.414	
>6 months	26.2%	10.3%	36.5%	45.0%	16.1%	0.001**	
Missing	1.9%	5.1%	1.2%	0.0%	1.6%	0.428	
**Relationship status**							
Single	55.3%	66.7%	48.2%	60.0%	56.5%	0.265	
In relationship	25.2%	17.9%	30.6%	20.0%	24.2%	0.438	
Separated/divorced/widow	18.9%	12.8%	21.2%	20.0%	19.4%	0.741	
Missing	0.5%	2.6%	0.0%	0.0%	0.0%	0.231	
**Education level**							
Less than HS	27.2%	23.1%	24.7%	35.0%	30.6%	0.662	
HS/GED	37.9%	48.7%	38.8%	30.0%	32.3%	0.342	
Some college	25.2%	23.1%	23.5%	30.0%	27.4%	0.891	
College+	8.7%	2.6%	12.9%	5.0%	8.1%	0.247	
Missing	1.0%	2.6%	0.0%	0.0%	1.6%	0.508	
**Age**							
Under 30	19.9%	25.6%	15.3%	30.0%	19.4%	0.358	
30–40	44.7%	43.6%	47.1%	40.0%	43.5%	0.936	
Over 40	32.5%	28.2%	37.6%	25.0%	30.6%	0.580	
Missing	2.9%	2.6%	0.0%	5.0%	6.5%	0.132	
**Employment status**							
Unemployed or under	86.4%	92.3%	91.8%	70.0%	80.6%	0.023*	
Employed full or PT	7.3%	0.0%	5.9%	20.0%	9.7%	0.035*	
Unknown/not reported	6.3%	7.7%	2.4%	10.0%	9.7%	0.259	
**Ever in foster care or adopted**							
No	80.1%	79.5%	80.0%	80.0%	80.6%	0.999	
Yes	18.0%	15.4%	20.0%	15.0%	17.7%	0.911	
Missing	1.9%	5.1%	0.0%	5.0%	1.6%	0.189	
**External support**							
Attended self-help group	47.6%	46.2%	45.9%	45.0%	51.6%	0.899	
Attended religious group	33.0%	20.5%	34.1%	30.0%	40.3%	0.223	
Attended recovery org	33.0%	35.9%	30.6%	20.0%	38.7%	0.421	
Attend any of the above	66.5%	61.5%	69.4%	55.0%	69.4%	0.538	
**Count of substance used past 90 days (up to 13 substances)**							
None	45.6%	41.0%	43.5%	50.0%	50.0%	0.773	
1–3	38.3%	35.9%	41.2%	35.0%	37.1%	0.914	
4 or More (max 8)	16.0%	23.1%	15.3%	15.0%	12.9%	0.585	
**# Days alcohol used during past 90 days**							
0 day	62.6%	61.5%	54.1%	75.0%	71.0%	0.122	
1–30 Days	25.2%	28.2%	25.9%	20.0%	24.2%	0.913	
>30 days	12.1%	10.3%	20.0%	5.0%	4.8%	0.028*	
**Trauma variable**	**Variable type (min-max)**	**All sample**	**No engagement**	**Session only**	**Healthcare only**	**Full engagement**	***p*** **-value**
N		206	39	85	20	62	
IPV (OAS)	Binary	0.82	0.79	0.74	0.85	0.92	0.050*
Abuse: emotional	Binary	0.74	0.77	0.73	0.7	0.76	0.921
Financial	Binary	0.83	0.74	0.86	0.85	0.85	0.403
Relationship	Binary	0.32	0.21	0.31	0.25	0.42	0.126
Other Events	Binary	0.04	0.05	0.01	0	0.1	0.065
Abuse: Physical	Cont (0–5)	2.42 (1.49)	1.97 (1.5)	2.66 (1.44)	1.8 (1.28)	2.56 (1.51)	0.019*
Abuse: Sexual	Cont (0–5)	1.68 (1.53)	1.49 (1.59)	1.81 (1.51)	1.45 (1.57)	1.71 (1.54)	0.637
Family	Cont (0–8)	3.92 (1.76)	3.62 (1.94)	4.15 (1.74)	3.4 (1.64)	3.95 (1.7)	0.222
Medical	Cont (0–4)	1.95 (1.18)	1.56 (1.35)	2.14 (1.11)	1.9 (1.29)	1.94 (1.08)	0.092
System	Cont (0–2)	1.18 (0.45)	1.08 (0.42)	1.24 (0.45)	1.15 (0.49)	1.19 (0.44)	0.323
Trauma Score	Cont (0–29)	13.9 (5.23)	12.28 (5.89)	14.65 (5.02)	12.35 (4.88)	14.4 (4.95)	0.049*

*SD in parentheses; Chi-square test of independence used for binary variables; ANOVA used for continuous variables;*

* *statistically significant at the 0.05 level;*

** *statistically significant at the 0.001 level*.

#### HIV/STI/HCV Risks

Table 2 presents findings from bivariate comparisons of HIV/STI/HCV risk behaviors (“no risky behaviors”, “any risk behaviors”, “no 6-month data”), on trauma, study controls and sociodemographic variables. Significant associations were identified between Engagement Type “None” (or not engaging in WORTH Transitions) and HIV/STI/HCV risks (*p* = 0.014). Significant associations were also identified between Age and HIV/STI/HCV risk behaviors (Under 30 years old, *p* = 0.014; Over 40 years old, *p* = 0.001). There was no significant variation in the accumulated Trauma Score across the three HIV/STI/HCV risk behavior outcomes examined (“No Risky Behaviors”, “Any Risky Behaviors”, “No 6-month Data”). The proportion of study participants who had any Engagement does however vary across risk behavior outcomes. Specifically, those in the “No Risky Behavior” category had the highest proportion of those who were engaged (94.7%), while those in the No 6-month data category had the lowest proportion (75.4%; *p* < 0.05). Moreover, the “No Risky Behavior” category had the highest proportion of participants who were 40 or older (55.3%), while the “Any Risky Behavior” category had the lowest proportion of participants who were 40 or older (14.75; *p* < 0.05).

**Table 2:** Descriptive statistics by HIV/STIs/HCV risks.

**Patient characteristics**	**No risky behaviors** **(*n* = 38)**	**Any risky behavior** **(*n* = 34)**	**No response** **(*n* = 134)**	***p*-value**
**Engagement type**				
None	5.3%	11.8%	24.6%	0.014*
Session only	47.4%	44.1%	38.8%	0.597
Healthcare only	10.5%	2.9%	11.2%	0.343
TC/PCP and session	36.8%	41.2%	25.4%	0.121
Trauma score	13.5 (5.5)	13.9 (5.3)	14.0 (5.1)	0.858
**Race/ethnicity**				
White	50.0%	58.8%	53.7%	0.754
Minority	50.0%	41.2%	45.5%	0.754
Missing	0.0%	0.0%	0.7%	0.763
**Homelessness**				
Not homeless	57.9%	70.6%	59.7%	0.458
Homeless	42.1%	29.4%	40.3%	0.458
**Depression**				
Severe	26.3%	23.5%	23.9%	0.963
None or moderate	71.1%	73.5%	73.1%	0.948
Missing	2.6%	2.9%	3.0%	0.993
**PTSD**				
Negative	21.1%	23.5%	14.9%	0.405
Positive	76.3%	76.5%	85.1%	0.301
Missing	2.6%	0.0%	0.0%	0.108
**Time since release**				
<1 month	28.9%	35.3%	18.7%	0.081
1–3 months	28.9%	20.6%	31.3%	0.468
3–6 months	15.8%	8.8%	23.1%	0.139
>6 months	26.3%	35.3%	23.9%	0.401
Missing	0.0%	0.0%	3.0%	0.334
**Relationship status**				
Single	42.1%	67.6%	56.0%	0.091
In relationship	34.2%	17.6%	24.6%	0.261
Separated/divorced/widow	23.7%	14.7%	18.7%	0.618
Missing	0.0%	0.0%	0.7%	0.763
**Education level**				
Less than HS	36.8%	17.6%	26.9%	0.186
HS/GED	36.8%	44.1%	36.6%	0.713
Some college	21.1%	23.5%	26.9%	0.743
College+	5.3%	14.7%	8.2%	0.343
Missing	0.0%	0.0%	1.5%	0.581
**Age**				
Under 30	5.3%	32.4%	20.9%	0.014*
30–40	34.2%	47.1%	47.0%	0.357
Over 40	55.3%	14.7%	30.6%	0.001**
Missing	5.3%	5.9%	1.5%	0.252
**Count of substance used (up to 13 substances)**				
None	42.1%	50.0%	45.5%	0.797
1–3	47.4%	29.4%	38.1%	0.292
4 or More (max 8)	10.5%	20.6%	16.4%	0.498
**Employment status**				
Unemployed or under	81.6%	82.4%	88.8%	0.389
Employed full or PT	13.2%	8.8%	5.2%	0.234
Unknown/not reported	5.3%	8.8%	6.0%	0.795
**Ever in foster care or adopted**				
No	76.3%	73.5%	82.8%	0.388
Yes	23.7%	23.5%	14.9%	0.301
Missing	0.0%	2.9%	2.2%	0.609
Had any external support	65.8%	64.7%	67.2%	0.959
**# Days alcohol used during past 90 days**				
0 day	63.2%	67.6%	61.2%	0.783
1–30 days	26.3%	20.6%	26.1%	0.791
>30 days	10.5%	11.8%	12.7%	0.935

* *statistically significant at the 0.05 level;*

** *statistically significant at the 0.001 level*.

### Longitudinal Multivariate Analyses

#### Engagement Type

As illustrated in Table 3, a 1-point increase in the trauma score is associated with 9.2% higher probability of engaging in session only (*p* < 0.05), and an 8.2% higher probability of engaging in all aspects of the study (session + healthcare) (*p* < 0.05), relative to those with no engagement. After controlling for all the potential confounders (in the adjusted model), these associations are no longer statistically significant, and become smaller in magnitude. In adjusted models, Black or indigenous women of color had a higher odds of engaging in sessions only and in the clinic only when compared with their white peers (*p* = 0.014).

**Table 3:** Longitudinal multivariate analysis trauma, psychopathology, and all other variables by engagement type.

**Covariate**	**Model 1a-U**				**Model 1a**			
	**Multinomial logistic regression model: unadjusted**				**Multinomial logistic regression model: adjusted**			
**No engagement vs. session only**	**RRR**	***p*-value**	**(95% CI)**		**RRR**	***p*-value**	**(95% CI)**	
Trauma score	1.092	0.020	1.014	1.177	1.029	0.574	0.930	1.139
Any external support (vs. none)					1.404	0.482	0.546	3.613
Minority (vs. non-minority or unknown)					3.575	0.014*	1.293	9.887
Severe depression (vs. none, moderate, or unknown)					1.632	0.373	0.556	4.789
PTSD positive (vs. negative or unknown)					4.804	0.012*	1.417	16.292
**Relationship status**								
Single					(referent category)			
In relationship					1.904	0.281	0.590	6.139
Separated/divorced/unknown					1.994	0.310	0.526	7.561
Ever foster/adopted (vs. never or unknown)					0.810	0.729	0.245	2.673
Full or PT employed (vs. not full or PT employed)					0.630	0.339	0.245	1.625
**Education level**								
Less than HS					(referent category)			
HS/GED					0.888	0.835	0.290	2.716
Some college					1.202	0.777	0.337	4.291
College+ or unknown					3.527	0.192	0.531	23.449
**Age category**								
Age: 18–29					(referent category)			
Age: 30–40					1.416	0.564	0.435	4.609
Age: >40 or unknown					1.552	0.496	0.438	5.506
**Time since release from prison**								
<3 months					(referent category)			
1–3 months					0.200	0.011*	0.058	0.690
3–6 months					0.346	0.142	0.084	1.427
>6 months or unknown					1.379	0.635	0.367	5.183
**Count of substance used (up to 14 substances)**								
None					(referent category)			
1–3					0.978	0.969	0.311	3.078
4 or more (max 9)					0.593	0.468	0.145	2.434
**# Days alcohol used during past 90 days**								
0 day					(referent category)			
1–30 days					0.760	0.651	0.232	2.492
>30 days					1.268	0.767	0.264	6.105
Constant	0.661	0.441	0.231	1.893	0.165	0.093	0.020	1.353
**Covariate**	**Multinomial logistic regression model: unadjusted**				**Multinomial logistic regression model: adjusted**			
**No engagement vs. healthcare only**	**RRR**	***p*** **-value**	**(95% CI)**		**RRR**	***p*** **-value**	**(95% CI)**	
Trauma score	1.002	0.963	0.906	1.110	0.970	0.680	0.838	1.122
Any external support (vs. none)					0.839	0.803	0.211	3.330
Minority (vs. non-minority or unknown)					8.298	0.004*	1.962	35.092
Severe depression (vs. none, moderate, or unknown)					2.059	0.353	0.449	9.454
PTSD positive (vs. negative or unknown)					2.244	0.323	0.451	11.168
**Relationship status**								
Single					(referent category)			
In relationship					1.117	0.894	0.218	5.715
Separated/divorced/unknown					2.779	0.283	0.430	17.957
Ever foster/adopted (vs. never or unknown)					0.599	0.582	0.096	3.717
Full or PT employed (vs. not full or PT employed)					1.460	0.469	0.525	4.062
**Education level**								
Less than HS					(referent category)			
HS/GED					0.237	0.073	0.049	1.144
Some college					0.781	0.760	0.159	3.831
College+ or unknown					0.439	0.569	0.026	7.465
Age category								
Age: 18–29					(referent category)			
Age: 30–40					0.754	0.730	0.153	3.729
Age: >40 or unknown					0.833	0.832	0.154	4.511
**Time since release from prison**								
<3 months					(referent category)			
1–3 months					1.005	0.996	0.120	8.389
3–6 months					3.143	0.306	0.351	28.138
>6 months or unknown					10.335	0.029*	1.270	84.096
**Count of substance used (up to 14 substances)**								
None					(referent category)			
1–3					0.929	0.930	0.184	4.705
4 or More (max 9)					1.866	0.552	0.239	14.561
**# Days alcohol used during past 90 days**								
0 day					(referent category)			
1–30 days					0.295	0.168	0.052	1.675
>30 days					0.142	0.191	0.008	2.643
Constant	0.498	0.316	0.127	1.948	0.103	0.128	0.005	1.929
**Covariate**	**Multinomial logistic regression model: unadjusted**				**Multinomial logistic regression model: adjusted**			
**No engage vs. session** **+** **TC**	**RRR**	***p*** **-value**	**(95% CI)**		**RRR**	***p*** **-value**	**(95% CI)**	
Trauma score	1.082	0.049	1.000	1.170	1.048	0.373	0.945	1.162
Any external support (vs. none)					1.513	0.405	0.571	4.012
Minority (vs. non-minority or unknown)					3.465	0.018*	1.236	9.710
Severe depression (vs. none, moderate, or unknown)					1.768	0.306	0.594	5.266
PTSD positive (vs. negative or unknown)					2.243	0.191	0.669	7.519
**Relationship status**								
Single					(referent category)			
In relationship					1.512	0.501	0.453	5.048
Separated/divorced/unknown					1.476	0.580	0.372	5.848
Ever foster/adopted (vs. never or unknown)					0.709	0.580	0.210	2.398
Full or PT employed (vs. not full or PT employed)					1.262	0.578	0.555	2.871
**Education level**								
Less than HS					(referent category)			
HS/GED					0.518	0.242	0.172	1.558
Some college					0.832	0.772	0.239	2.897
College+ or unknown					1.522	0.672	0.218	10.618
**Age category**								
Age: 18–29					(referent category)			
Age: 30–40					1.232	0.732	0.374	4.064
Age: >40 or unknown					1.427	0.580	0.405	5.031
**Time since release from prison**								
<3 months					(referent category)			
1–3 months					0.957	0.945	0.271	3.374
3–6 months					1.402	0.647	0.331	5.942
>6 months or Unknown					1.356	0.683	0.315	5.846
**Count of substance used (up to 13 substances)**								
None					(referent category)			
1–3					1.134	0.832	0.353	3.640
4 or more (max 8)					0.740	0.676	0.180	3.040
**# Days alcohol used during past 90 days**								
0 day					(referent category)			
1–30 days					0.504	0.264	0.151	1.677
>30 days					0.210	0.107	0.031	1.399
Constant	0.554	0.297	0.183	1.681	0.161	0.087	0.020	1.305

* *statistically significant at the 0.05 level*.

Having PTSD symptomology was significantly associated with participating in sessions only (*p* = 0.012), whereas being out of jail for a longer period of time (>6 months) was significantly associated with attending the Transition Clinic only (*p* = 0.029), as compared with those who did not participate in any study activities after enrollment/baseline.

#### HIV/STI/HCV Risks

And finally, Table 4 presents the adjusted and unadjusted longitudinal multivariate models examining associations between baseline trauma, mental health, and engagement type on HIV/STI/HCV outcomes/having self-reported HIV/STI/HCV. There was no statistically significant association—either in the adjusted or the unadjusted models—between the HIV/STIs/HCV risk behaviors and the key explanatory variables: baseline trauma, depressive and PTSD symptomology, and treatment engagement. Similarly, significant risks were not identified between the number of days alcohol was used during past 90 days or the count of substance used in the past 90 days reported at baseline and HIV/STI/HCV risk outcomes. Positive HIV/STIs/HCV outcomes were solely associated with being in a relationship (lowered risk: relative risk ratio = 0.188; *p* = 0.023).

**Table 4:** Longitudinal multivariate analysis trauma, psychopathology, and all other variables by HIV/STIs/HCV risk behaviors.

**Covariate**	**Model 2a-U**				**Model 2a**			
	**No risky behaviors vs. any risky behaviors (no interaction): unadjusted**				**No risky behaviors vs. any risky behaviors (no interaction): adjusted**			
	**RRR**	***p*-value**	**95% CI**		**RRR**	***p*-value**	**95% CI**	
**Engagement type**								
None	(referent category)				(referent category)			
Session only	0.400	0.332	0.063	2.543	0.240	0.214	0.025	2.279
Healthcare only	0.124	0.140	0.008	1.986	0.062	0.086	0.003	1.487
Full engagement	0.481	0.443	0.074	3.118	0.422	0.443	0.047	3.823
Trauma score	1.014	0.759	0.925	1.112	1.074	0.299	0.939	1.229
Any external support (vs. none)					1.039	0.952	0.298	3.622
Minority (vs. non-minority or unknown)					0.757	0.656	0.222	2.580
Severe depression (vs. none, moderate, or unknown)					0.821	0.770	0.220	3.072
PTSD positive (vs. negative or unknown)					2.137	0.345	0.442	10.329
**Relationship status**								
Single					(referent category)			
In relationship					0.188	0.023	0.044	0.796
Separated/divorced/unknown					0.282	0.133	0.054	1.470
Ever foster/adopted (vs. never or unknown)					0.873	0.851	0.211	3.609
Full or PT employed (vs. not full or PT employed)					1.168	0.754	0.443	3.076
**Education level**								
Less than HS					(referent category)			
HS/GED					3.809	0.069	0.899	16.141
Some college					3.435	0.136	0.678	17.417
Less than HS					9.960	0.054	0.959	103.466
**Age category**								
Age: 18–29					(referent category)			
Age: 30–40					0.159	0.052	0.025	1.016
Age: >40 or unknown					0.025	0.000**	0.004	0.178
**Time since release from prison**								
<3 months					(referent category)			
1–3 months					0.457	0.331	0.094	2.216
3–6 months					0.684	0.702	0.098	4.774
>6 months or unknown					1.991	0.359	0.457	8.677
**Count of substance used (up to 13 substances)**								
None					(referent category)			
1–3					0.426	0.252	0.099	1.834
4 or more (max 8)					1.187	0.865	0.164	8.577
**# Days alcohol used during past 90 days**								
0 day								
1–30 days					1.785	0.476	0.362	8.795
>30 days					2.896	0.309	0.373	22.469
Constant	1.700	0.601	0.232	12.435	5.843	0.289	0.223	152.928
**Covariate**	**No risky behaviors vs. no 6-mo data (no interaction): unadjusted**				**No risky behaviors vs. no 6-mo data (no interaction): adjusted**			
	**RRR**	***p*-value**	**95% CI**		**RRR**	***p*-value**	**95% CI**	
**Engagement type**								
None	(referent category)				(referent category)			
Session only	0.157	0.019	0.033	0.734	0.075	0.006*	0.012	0.481
Healthcare only	0.224	0.105	0.037	1.366	0.101	0.035*	0.012	0.846
Full engagement	0.133	0.012	0.028	0.641	0.069	0.004*	0.011	0.430
Trauma score	1.041	0.277	0.968	1.121	1.084	0.121	0.979	1.201
Any external support (vs. none)					1.167	0.760	0.434	3.142
Minority (vs. non-minority or unknown)					1.292	0.589	0.509	3.278
Severe depression (vs. none, moderate, or unknown)					0.872	0.788	0.321	2.372
PTSD positive (vs. negative or unknown)					3.722	0.040*	1.060	13.066
**Relationship status**								
Single					(referent category)			
In relationship					0.434	0.135	0.145	1.297
Separated/divorced/unknown					0.440	0.192	0.128	1.512
Ever foster/adopted (vs. never or unknown)					0.402	0.102	0.135	1.198
Full or PT employed (vs. not full or PT employed)					0.887	0.758	0.414	1.902
**Education level**								
Less than HS					(referent category)			
HS/GED					1.625	0.355	0.581	4.548
Some college					2.092	0.221	0.642	6.811
College + or unknown					5.913	0.075	0.833	41.949
**Age category**								
Age: 18–29					(referent category)			
Age: 30–40					0.311	0.180	0.056	1.713
Age: >40 or Unknown					0.079	0.003*	0.014	0.433
**Time since release from prison**								
<3 months					(referent category)			
1–3 months					1.280	0.691	0.378	4.333
3–6 months					3.519	0.088	0.829	14.932
>6 months or Unknown					2.569	0.127	0.764	8.640
**Count of substance used (up to 13 substances)**								
None					(referent category)			
1–3					0.678	0.505	0.216	2.127
4 or More (max 8)					1.370	0.706	0.266	7.047
**# Days alcohol used during past 90 days**								
0 day								
1–30 days					1.409	0.580	0.418	4.744
>30 days					1.915	0.445	0.362	10.125
Constant	10.233	0.006	1.970	53.142	11.532	0.076	0.774	171.787

* *statistically significant at the 0.05 level;*

** *statistically significant at the 0.001 level*.

When comparing those who did not report risk outcomes to those were lost to follow up, those who participated in the study in any way (sessions only: relative risk ratio = 0.075, *p* = 0.006; Transitions Clinic: relative risk ratio = 0.101, *p* = 0.035; WORTH sessions and Transitions Clinic: relative risk ratio = 0.069; *p* = 0.004) had more than 90% lower probability of being lost to follow up. In addition, those participants who were 40 or older were significantly less likely to be in the lost to follow up category (relative risk ratio = 0.079 or more than 90% lower probability; *p* = 0.003), suggesting that the sample attrition was associated with younger patient age. Conversely, relative to those who did not engage in HIV/STI/HCV risky behaviors, those with PTSD symptoms (*p* = 0.040) were associated with more than 3-fold increase in the probability of being lost to follow up (relative risk ratio = 3.722).

## Discussion

This study presents multiple important findings related to associations between trauma, mental health, type of treatment engagement, and HIV/STI/HCV risk outcomes among women recently released from incarceration. It also draws attention to what appears to be a highly complex relationship between trauma, PTSD-specific mental health distress, engagement in HIV/STI/HCV prevention services, and HIV/STI/HCV risks. First, although, women transitioning from incarceration have been characterized as among the hardest to engage, study participants on the whole exhibited high rates of engagement in WORTH Transitions activities and non-WORTH Transitions self-help programs (e.g., NA, AA, spiritual counseling) despite simultaneously experiencing acute levels of trauma and PTSD symptomatology. Contrary to earlier findings (30), PTSD (vs. depressive) symptomology served as an “engagement catalyst”. Specifically, having PSTD symptomology was associated with a significantly higher odds of engaging in peer-led individual and/or group WORTH sessions as was being a Black or Indigenous woman of color (BIWOC). Interestingly, PTSD symptomology was also associated with a significantly higher odds of being lost to follow up in the longitudinal model.

The fact that those with PTSD symptomology turned toward, rather than away from, peer-led activities (at least initially) lends additional support to preliminary findings regarding the efficacy of peer engagement (57). It also underscores the continuing utility of session-based HIV prevention interventions/activities like WORTH Transitions, as compared to asynchronous activities increasingly lauded as less expensive and less time consuming (58–60). Study findings instead suggest that interventions like WORTH Transitions remain highly salient in the arsenal of HIV/STI/HCV prevention interventions. This may be especially true for BIWOC populations. As noted, BIWOC were significantly more likely to engage in all study activities (sessions only, healthcare only, sessions + healthcare), standing in contrast to prior findings categorizing this sub-group as being especially “hard to reach” (31, 61). Although additional research is needed, peer-based prevention interventions like WORTH Transitions may serve to mitigate treatment hesitancy identified among BIWOC (62) and/or challenge the existing narrative of treatment resistance.

These encouraging findings were not true for all participants however as results make clear that a subset of women most at risk for HIV/STI/HCV were less likely to engage and be retained. Specifically, since findings identified significant associations between “not engaging” and HIV/STI/HCV risks/outcomes and PTSD symptomology and a more than 3-fold increase in the probability of being lost to follow up (relative risk ratio = 3.722), additional research is needed to identify which subset of recently released women are not fully reached by the pairing of these two evidence-based interventions (WORTH Transitions) as it is currently designed. As an initial next step, we recommend that qualitative and/or multimethod studies be conducted to identify the total number of group sessions that may be tolerable (or non-exacerbating) for transitioning women exhibiting severe PTSD symptomology. Since findings from this current study also makes clear that significant within-group differences exist among women transitioning from carceral settings, we recommend that future studies utilize the lens of intersectionality (63) to examine trauma and mental health distress, sociodemographic characteristics (race/ethnicity, age, etc.), HIV/STI/HCV risks and engagement in HIV/STI/HCV prevention services in this population. Also through the lens of intersectionality, we recommend that research be conducted with the goal of more finely attuning peer-led HIV/STI/HCV risk prevention interventions by type to highly traumatized women emerging from jails and prisons based on their sociodemographic characteristics and trauma and PTSD symptomology. Given the findings from this current study, it could be the case, for example, that highly traumatized older BIPOC women currently experiencing PTSD symptoms may be more responsive to lengthier peer-led approaches delivered in a group setting, while those who are younger may require individualized peer delivered approaches, regular pre-, during-, or post-group check-ins, and/or groups that are shorter in duration. Developing a typology of peer-led interventions, matched to both trauma and PTSD phenotype and sociodemographic characteristics will significantly advance efforts to prevent new transmissions among this highly vulnerable group who remain at the forefront of HIV/STI/HCV risk. This also has implications in terms of trauma-informed care (TIC) and is consistent with prior calls for patient-centered TIC approaches to mitigate HIV/STI/HCV and other health and behavioral health risks during re-entry (64).

Second, consistent with extant literature (65), only a subset of trauma types were heavily endorsed. Unsurprisingly, high rates of social and structural forms of trauma (e.g., financial-, system-, and medical-) were identified. While peer review literature is replete with references to financial-, system-, and/or medical-related harm experienced by women in the criminal legal system pre-, during and post incarceration (66), to our knowledge, only one peer reviewed manuscript has examined associations between “social and/or structural trauma” on HIV/STI/HCV risks among recently released women (67). None to date have examined how these forms of trauma may impact the ways in which women transitioning from incarcerated settings (or women in the criminal legal system as a whole) may engage in and/or are retained in HIV/STI/HCV prevention interventions. Additional research is needed to fill this gap. We specifically recommend that future researchers examine how social and structural trauma may be proximally or distally associated with PTSD-related mental health distress, engagement, and/or HIV/STI/HCV outcomes. In addition, while the efficacy of trauma-informed care for incarcerated populations has been well documented in scholarly literature (68), far less is known regarding how (1) to target recently released women in particular (29); and (2) HIV/STI/HCV prevention services and trauma-informed services should be differently structured and/or delivered based on social and structural trauma histories.

Third, results suggest that longer windows of time since release are associated with significantly higher odds of engagement in Transition Clinic health services and as such may serve as a “critical time” for optimal engagement. While these findings are on the whole unsurprising given the preponderance of peer reviewed literature pointing to the need for targeted support during this especially vulnerable period of transition and risk (69), they nevertheless speak to the importance of optimally timed interventions. Building on this body of literature, the Transitions Clinic program specifically aims to reach individuals within 2 weeks of release and includes peer in-reach to jails and prisons prior to release (29). This study offers insight on future directions of research in order to deepen scholarly and practitioner understanding of how these interventions should be structured and optimally timed. It may be that more flexibility is needed in addressing women's varied mental health, physical health, and structural priorities, depending on their level of mental health distress and PTSD symptomology as they transition from carceral settings.

Lastly, the findings from this study suggest that neither trauma nor mental health symptomology was associated with higher amounts of HIV/STI/HCV risks. Conversely, being in a relationship was found to be protective. While a small handful of peer reviewed studies have pointed to a potentially protective role of having a committed partner (25), HIV/STI/HCV risks have instead overwhelmingly been attributed to risky male sexual partners (70). We therefore recommend that additional research be conducted with recently released women to determine if and/or how the level of protection offered may change over time. For example, given afore referenced findings pointing to the saliency of the window post release, it may be possible that the more time recently released women spend with their partners, partner-related HIV/STI/HCV risks may increase.

Additional qualitative quantitative, and mixed methods studies from this data will explore several of these themes including depression, trauma and other factors associated with differences in levels of engagement and the implementation, fidelity, and adherence of the study.

## Strengths and Limitations

Strengths of this study includes its novel use of two evidence-based interventions, WORTH (and E-WORTH) which has been deemed highly efficacious in preventing HIV/STIs/HCV among women in the criminal legal system, and Transitions Clinic which effectively uses peers to increase engagement.

Limitations include sample size limitations impacting the level of power needed to detect true effect sizes in some longitudinal models and the use of self-reported HIV/STIs/HCV risk data and biological outcomes. Self-reported data has proven reliable in research studies however (71) and respondents across studies have demonstrated high recall for HIV and health-related outcomes (72). In addition, having an English-speaking requirement may have limited the number of Latina participants who were less proficient in English.

## Conclusion

This manuscript presents important findings regarding the impact of trauma and mental health symptoms on HIV/STI/HCV engagement in a highly efficacious HIV/STI/HCV prevention intervention among recently released women. Results underscore the extent to which this group may be health seeking, desirous of support and reachable by services that are appropriately tailored, contradicting prior research. Through this lens, this study also provides insight regarding how to best tailor HIV/STI/HCV prevention and intervention services for this highly vulnerable population of women in the U.S. criminal legal system who remain among the most vulnerable to new transmission.

## Data Availability Statement

The raw data supporting the conclusions of this article will be made available by the authors, without undue reservation.

## Ethics Statement

The studies involving human participants were reviewed and approved by University of Rochester Institutional Review Board. The patients/participants provided their written informed consent to participate in this study.

## Author Contributions

KJ served as primary author in preparing the manuscript and contributed to research design. DM, TH, and LP edited extensively and contributed to research design. AN, AL, ZS, RC, JW, and RJ contributed significantly to the literature review, outline, and introduction. DM ran all analyses. All other authors provided critical review. All authors contributed to the article and approved the submitted version.

## Funding

The study was funded by a Substance Abuse and Mental Health Services Administration (SAMHSA) principal investigator DSM (grant #1 H79 TI080055-01). The funders had no role in study design, data collection and analysis, decision to publish, or preparation of the manuscript.

## Conflict of Interest

The authors declare that the research was conducted in the absence of any commercial or financial relationships that could be construed as a potential conflict of interest.

## Publisher's Note

All claims expressed in this article are solely those of the authors and do not necessarily represent those of their affiliated organizations, or those of the publisher, the editors and the reviewers. Any product that may be evaluated in this article, or claim that may be made by its manufacturer, is not guaranteed or endorsed by the publisher.
